# Field survey of major infectious and reproductive diseases responsible for mortality and productivity losses of ruminants amongst Nigerian Fulani pastoralists

**DOI:** 10.12688/gatesopenres.13164.1

**Published:** 2020-10-19

**Authors:** Muhammed B. Bolajoko, Franciscus Van Gool, Andew R. Peters, Jeimmy Suarez Martinez, Ciara J. Vance, Baptiste Dungu

**Affiliations:** 1 Veterinary Public Health and Preventive Medicine, National Veterinary Research Institute, Vom, Nigeria; 2 Excelvet Consultants, Mandelieu, France; 3 Supporting Evidence Based Interventions (SEBI), University of Edinburgh, Edinburgh, EH25 9RG, UK; 4 Onderstepoort Biological Products, Pretoria, South Africa

**Keywords:** Nigeria, ruminants, livestock, disease, productivity, mortality

## Abstract

**Background:** Animal disease constitutes a major hurdle to improved livelihoods in rural Nigeria through the challenges of loss of productivity, livestock morbidity and mortality including reproductive losses. In order to design and implement impactful interventions, baseline data on the causes of such losses are needed. Therefore, the objective of the present study was to carry out targeted field surveys, including interviews with ruminant farmers, veterinary professionals and other stakeholders in livestock farming to establish the main causes of disease and mortality including abortions in cattle and small ruminants (SR).

**Methods:** Northern Nigeria was selected because the majority of the nation’s ruminants belong to pastoralists who are primarily resident in this region. Seven states; Bauchi, Kaduna, Kano, Nasarawa, Niger, Sokoto and Zamfara states were surveyed. The responses were collated and a comprehensive descriptive analysis was carried out.

**Results:** Average cattle herd sizes ranged from 28 in Zamfara to 103 in Nasarawa; and from 27 in Kano to 128 in Sokoto for SR. In cattle, Trypanosomosis (with 4.27% mortality rate), foot and mouth disease (3.81%), nutritional insufficiency (1.93%) and contagious bovine pleuropneumonia (CBPP; 1.44%) were the top four diseases/health problems that resulted in the highest mortality due to diseases within each state surveyed. For SR, trypanosomosis (with 6.85% mortality rate), Peste des Petits Ruminants (4.99%), orf (3.06%), foot rot (2.97%) and foot and mouth disease (2.94%) were the most important diseases responsible for the highest number of mortalities and culling for disease.

**Conclusions: **The study revealed that there are significant losses via mortalities due to the occurrence of disease amongst the ruminant populations countrywide, as evidenced by the high overall mortality rates of both cattle (15.3%) and small ruminants (30.9%) from various diseases. Also, reproductive losses of 8.7% and 16.6% in cattle and SR, respectively, were recorded amongst the farmers involved.

## Introduction

Positioned as the most important national sector of the economy after oil, Nigerian agriculture is still largely under-developed, with significant regional diversity across the country ( Watts, 2006; Wikipedia). Analysis of the sector is inhibited by a paucity of data on its overall economic performance. However, the available statistics provide a subjective and broad overview of development in agriculture upon which some broad generalisations can be made about its role in economic development and structural change in Nigeria. Livestock as a sub-sector of agriculture contributes significantly to the protein needs and economy of rural and urban communities ( Bradford, 1999) and the nation, accounting for one third of Nigeria’s agricultural Gross Domestic Product (GDP), providing income, employment, food, energy, manure, fuel and transport ( Anon, 2006; Inter Academy Council, 2004).

Increasingly, animal production trends are influenced by strong demand-driven factors such as population growth, urbanization, income growth and changing customer preferences, which are of two categories: (1) a modern demand driven and capital-intensive non-ruminant (swine and avian) sector and (2) the traditional resource-driven (constrained) and labour-intensive ruminant (cattle, sheep and goats) sector ( Devendra, 2002; Devendra, 2007).

Of the numerous challenges that Nigerian animal production faces preventing optimal productivity, one of the most prominent is animal disease. In addition to such challenges, the bulk of the livestock farmers are pastoralists and rural farmers and to date, they have largely resisted transfer from traditional to modern production methods ( Blench, 1999). Most farmed livestock, especially ruminants, are managed by the Fulanis who have largely retained the transhumance pastoral system ( Blench, 1990; Blench, 1999; Elelu *et al*., 2016; Lawal-Adebowale, 2012).

Livestock production is thus constantly under threat by diseases that ultimately reduce productivity and revenue generation ( MacRae *et al.,* 2005). Endemic animal diseases such as helminthiasis, contagious bovine pleuropneumonia (CBPP), foot and mouth disease (FMD) brucellosis, mastitis, peste des petits ruminants (PPR), and many others have devastating impacts upon the livestock sector, leading to losses of hundreds of millions of dollars every year in developing economies like Nigeria ( Bamaiyi, 2012; Bhat *et al.,* 2012). For example, Brucellosis alone in sheep and goats in Borno and Yobe states was estimated to cost the Nigerian economy USD 3.2 million annually ( Brisibe *et al*., 1996).

Unfortunately, the limited availability of objective and valid data on the major health challenges leading to losses in ruminant production in Nigeria and other sub-Saharan economies has always constituted a major bottleneck to achieving meaningful and quantifiable interventions that could contribute to improved livestock health and productivity. The *Supporting Evidence Based Interventions* (SEBI) initiative was established in 2016 at the University of Edinburgh UK with the main objective of producing better data on livestock health and productivity from lower and middle-income countries (LMICs). Specifically, one of the first SEBI programmes was to quantify mortality rates of cattle and small ruminants and major causes of mortality in three countries, Nigeria, Tanzania and Ethiopia with the aim of using mortality as an indicator of the animal health status of the respective country. The present study was therefore designed to support and expand the available data and to establish baselines for mortality and reproductive losses and their causes in cattle and small ruminants (SR) of specific regions of Nigeria. This was achieved by carrying out targeted epidemiological field studies, including interviews with ruminant farmers and veterinary professionals involved in livestock farming, to establish the main causes of mortality (including culling for disease) and abortions in cattle and SR. The information thus collected could potentially assist in identifying interventions to achieve the desired reductions in mortality and reproductive losses in cattle and SR, thus boosting productivity.

## Methods

Mortality rate was defined as the percentage of a population of animals dying or that were culled for a specific disease over a one-year period from September 2017 to August 2018.

### Ethics and consent

No animals were involved in this work. Written ethical approval for this questionnaire-based study was obtained from the Ethics Committee of the National Veterinary Research Institute, Vom, Nigeria (NVRI AEC Ref No.: AEC/03/90/20) and signed off on March 20th 2017. Verbal informed consent was obtained from each participant prior to commencement of the survey. They were informed that their participation was voluntary, that their responses would be kept confidential and no names or identifying information linking them to the survey would be disclosed. In addition, they were assured that they were free to terminate their participation at any time. The use of verbal consent was approved by the ethics committee and each participant signed against their name to confirm that they had understood the aims of the survey and that they agreed to participate.

### Study areas

**Figure 1.**
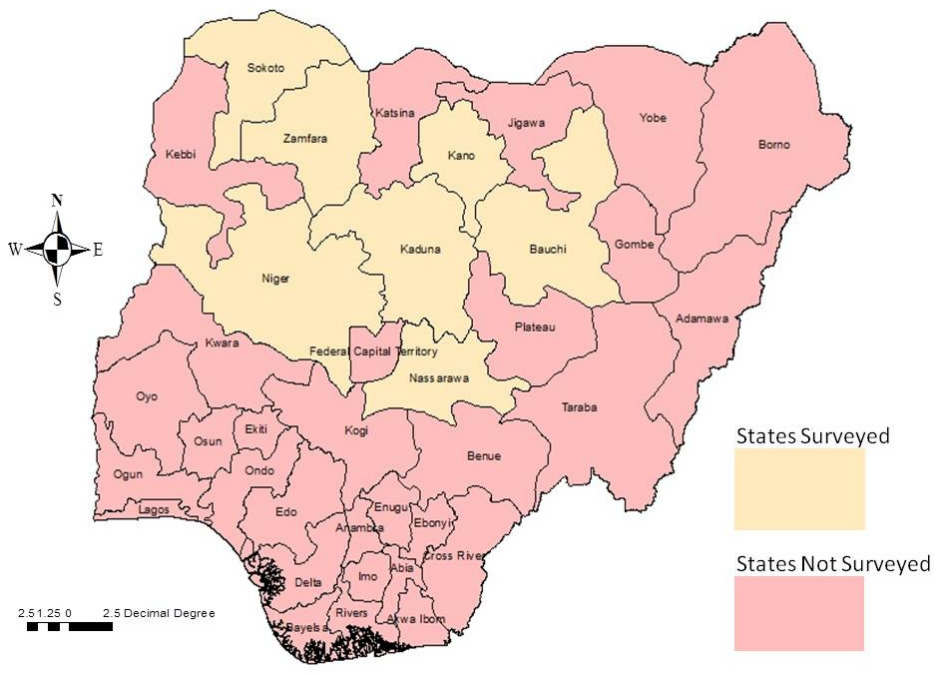
Map of Nigeria indicating the surveyed states from the remaining states of the federation.

The northern part of Nigeria was selected because the majority of the nation’s ruminants are owned by pastoralists who are primarily resident in this region of the country. A total of seven states; Bauchi, Kaduna, Kano, Nasarawa, Niger, Sokoto and Zamfara were surveyed as representative of the northern area, taking into consideration logistical, accessibility and security issues (see Figure 1). The regional agro-climates and reported human populations for each of the states are shown in Table 1.

**Table 1.** List of Nigerian states where the study was conducted.

State	Agro-climate	Source	Human population ( NASS, 2011)
Bauchi	Spans Sudan savannah (south) and Sahel savannah (north) vegetation zones	https://www.nigeriagalleria.com/Nigeria/States_Nigeria/Bauchi_State.html	4,653,066
Kaduna	Sudan savannah type	https://www.nigeriagalleria.com/Nigeria/States_Nigeria/Kaduna/Kaduna_State.html	6,113,503
Kano	Tropical savannah	https://www.nigeriagalleria.com/Nigeria/States_Nigeria/Kano/Brief-History-of-Kano.html	9,401,288
Nasarawa	Tropical with moderate rainfall. Mainly agricultural land	https://www.nigeriagalleria.com/Nigeria/States_Nigeria/Nasarawa_State.html	1,869,377
Niger	Largest Nigerian state by land area	https://www.nigeriagalleria.com/Nigeria/States_Nigeria/Niger/Niger_State.html	3,954,772
Sokoto	In dry Sahel, surrounded by sandy savannah and isolated hills	https://www.nigeriagalleria.com/Nigeria/States_Nigeria/Sokoto/Sokoto_State.html	3,702,676
Zamfara	Tropical climate	https://www.nigeriagalleria.com/Nigeria/States_Nigeria/Zamfara/Zamfara_State.html	3,278,873

### Methodology for data collection

*Field survey: group discussion with farmers:* Rapid rural appraisal (RRA) and/or participatory rural appraisal (PRA) methods ( Catley *et al*., 2011) in the form of open-ended group discussions were conducted with the designated farmer respondents in each state surveyed as described by Bolajoko *et al*. (2011) and Elelu *et al*. (2016). These were carried out to obtain the initial views of the farmers and to form baseline information about their personal experiences of their animals’ health status. Where necessary, the RRA and PRA were employed for further data collection and triangulation to clarify any unclear information collected during the survey. The data and information sourced during the discussions were recorded as non-numeric and non-categorical testimonies, explanations and interpretations.

*Field survey: questionnaire-based survey*: After completing the open-ended discussion with the farmer respondents in each of the seven states, a cross sectional questionnaire-based survey was then undertaken. The period surveyed was one year starting in September 2016. A total of 336 cattle farmers and 291 SR (sheep and goat) farmers participated in the study (see Table 2 and Table 3). These corresponded to a total sample of cattle and SR of 24,241 and 17,129, respectively ( Table 2 and Table 3).

**Table 2.** Distribution of respondent cattle farmers, total cattle population ( FMARD, 2010) and the numbers surveyed in the seven states surveyed.

States	Total cattle population in each state	Distribution of respondent farmers	Total cattle per state included in survey	Average size of herds included in survey
**Bauchi**	532,055	54	3,426	63
**Kaduna**	655,382	56	4,142	74
**Kano**	546,303	26	1,693	65
**Nasarawa**	1,312,481	47	4,859	103
**Niger**	221,473	55	4,102	75
**Sokoto**	392,872	55	4,819	88
**Zamfara**	3,175,050	43	1,200	28
**TOTAL**	6,835,616	336	24,241	-

**Table 3.** Distribution of small ruminant respondent farmers, total small ruminant population ( FMARD, 2010) and the numbers surveyed in the seven states surveyed.

States	Total small ruminant population in each state	Distribution of respondent farmers between states	Total small ruminant per state included in survey	Average flock size included in survey
**Bauchi**	542,485	51	2,094	41
**Kaduna**	3,028,059	27	1,066	39
**Kano**	6,673,970	25	666	27
**Nasarawa**	2,293,797	48	2,169	45
**Niger**	1,958,735	38	2,549	67
**Sokoto**	1,991,905	55	7,065	128
**Zamfara**	10,335,364	47	1,520	32
**TOTAL**	26,824,315	291	17,129	-

The questionnaires were structured in two sections: The first covered demographic data such as age, address, duration of ownership of ruminants and species of ruminants kept. The second part focused on disease, mortality and reproductive losses. Section 2 covered questions on specific diseases. A list of diseases and other conditions had been compiled on the basis of an earlier literature review conducted by the present authors on the relative prevalence of ruminant diseases in Nigeria. During the interviews the farmers were asked about their own experience of the occurrence of the specific conditions and their outcomes with the help of written descriptions of each condition together with photographs of typical signs. The same list of conditions was used to populate the data in Table 3– Table 6.

**Table 4.** Summary of the causes of mortality and culling of cattle in seven Nigerian states.

Ranking by farmers	Ranking by key informants	Disease identified from farmer survey	Number of cases	Percentage of total cattle surveyed	Percentage of all cattle mortalities
1	1	Trypanosomosis	1034	4.27	27.9
2	2	FMD	923	3.81	24.9
3	4	Inadequate nutrition	468	1.93	12.6
4		CBPP	348	1.44	9.4
5		Unspecified health problems	344	1.41	9.3
6		Sudden death	215	0.89	5.8
7	3	Lumpy skin disease	115	0.47	3.1
8	6	Endoparasites	103	0.42	2.8
9		Injuries	97	0.40	2.6
10	5	Pasteurellosis	53	0.22	1.4
**Total**			3700	15.30	100.0
**Total in** **survey**			24,241		

FMD, foot and mouth disease; CBPP, contagious bovine pleuropneumonia.

**Table 5.** Summary of the causes of mortality and culling of SR in seven Nigerian states.

Ranking by farmers	Ranking by key informants	Disease identified from farmer survey	Number of cases	Percentage of total SR surveyed	Percentage of all SR mortalities
1		Trypanosomosis	1174	6.85	22.2
2	1	PPR	854	4.99	16.1
3	3	Orf	524	3.06	9.9
4		Foot rot	509	2.97	9.6
5		FMD	504	2.94	9.5
6		Unspecified health problems	347	2.03	6.5
7	4	CCPP	313	1.83	5.9
8		Sudden death	299	1.75	5.6
9		Pasteurellosis	220	1.28	4.2
10		Injuries	200	1.17	3.8
11	5	Inadequate nutrition	179	1.05	3.4
12		Endoparasites	174	1.02	3.3
	2	Ectoparasites including fly strike			
	6	Sheep and goat pox			
Total			5297	30.93	100.0
Total in survey			17,129		

SR, small ruminants; PPR, peste des petits ruminants; FMD, foot and mouth disease; CCPP, contagious bovine pleuropneumonia.

**Table 6.** Summary of causes of abortion and other reproductive losses cases in the cattle-study population.

Putative cause of abortion / loss	Number of cases	Percentage of total pregnant cattle surveyed	Percentage of abortion cases
Brucellosis	515	3.61	41.4
Late abortions (> 5 months) due to unknown causes	246	1.72	19.8
Associated with FMD	239	1.68	19.2
Suspected early abortions (< 5 months)	117	0.82	9.4
Associated with heat stress	65	0.46	5.2
Associated with poor nutrition	62	0.43	5.0
**Total**	**1,244**	**8.72**	**100.0**
Total pregnant animals in survey	14,266		

FMD, foot and mouth disease.

The surveys were carried out by MBB (first author). The participants were initially contacted through their community leaders and the head of the farmers’ association in each state. The details of the study were provided in the letters written to these Heads in order for them to explain the main points to the farmers. Interested farmers were then invited to the initial group discussion with MBB. For this group discussion in each state, there was an initial brief introduction to the purpose of the study, followed by administration of the questionnaire. Thereafter, smaller group discussions were conducted each with 8–10 interested farmers. The farmers’ responses were documented during these sessions each of which lasted from 15 – 45 minutes. No specific checklist was used to guide the group discussion, but rather the questions raised during the introductory sessions were used to guide the direction of the further discussions.

*Consultation with key informants:* Questionnaires were sent by email to a purposively selected ( Thrusfield, 2018) cohort of field veterinarians, in both private and state employment and researchers actively engaged with ruminant farmers across the country. The questions were focused on the recognised major infectious and reproductive diseases of ruminants. The 16 respondents were asked to rank each condition by their perception of impact. Open-ended interviews and discussions were also carried out by MBB with these stakeholders on the same subject matter as the questionnaires. Their responses were recorded and documented. Each respondent session lasted from 20 – 40 minutes.

### Analyses

The responses from farmers and key informants were collated and entered into a specific database (Excel® 2016). The numbers of sick animals culled or sold by the farmers due to poor health were included in the computation of losses for mortality. A comprehensive descriptive analysis was then carried out using Microsoft Excel® 2016. Overall mortality rates were calculated by summing the total numbers of deaths / culls and dividing by the total numbers of animals on the holdings in the survey period. Disease specific mortality rates were calculated similarly by dividing the numbers of animals dying of the specified disease by the total number of animals on that holding.

## Results

The distribution of cattle farmers included in the study is shown in Table 2. Zamfara is the most populous state for cattle with more than 3 million head recorded while Niger had the lowest at around 220,000. The herd size of the cattle farms involved in the study ranged from 28 in Zamfara state to 103 in Nasarawa state ( Table 2). The distribution of SR farmers included in the study is shown in Table 3. Zamfara again is the most populous state for SR at more than 10 million while Bauchi has the smallest number estimated at around 540,000 ( Table 3). The average flock size for SR ranged from 27 in Kano state to 128 in Sokoto state ( Table 3).

The total number of cattle dying or culled over the survey period was 3,700 of the total 24,241 in the survey, representing a mortality rate of 15.3% ( Table 4; Bolajoko, 2020). The four most common causes of mortality or culling (disease-related disposal) were 1. trypanosomosis (4.27%), 2. foot and mouth disease (3.81%), 3. nutritional deficiency / starvation (1.93%) and 4. CBPP (1.44%) respectively ( Table 4). Other causes are listed in Table 4 and ranked according to the farmers’ responses. The ranking of the top six diseases by the key informants is also shown in Table 4 and corresponded well with the data from the farmers.

The total number of SR dying or culled over the survey period was 5,297 of the total 17,129 in the survey, representing a mortality rate of 30.9% ( Table 5). The five most common causes of mortality or disease-related disposal were 1. trypanosomosis (6.85%), 2. PPR (4.99%), 3. Orf (3.06%) 4. foot rot (2.97%) and 5. FMD (2.94%), respectively. Other causes are listed in Table 5, ranked according to the farmers’ responses. The ranking of the top six SR diseases by the key informants is also shown in Table 5 and while there is some agreement, the key informants did not rank trypanosomosis in SR, but ranked both ectoparasitism and CCPP very highly.

### Losses due to abortion in cattle and SR

The total number of reproductive losses in cattle during the survey period was 1,244 of the total 14,266 pregnant animals reported in the survey, representing a rate of 8.72% (see Table 6). Almost half (515; 3.61%) were thought to be due to brucellosis, with FMD another significant cause (239; 1.68%), the rest being due to a variety of other causes.

The total number of reproductive losses in SR during the survey period was 1,448 of the total 8,725 pregnant animals reported in the survey, representing a rate of 16.6% (see Table 7). Almost half (664; 7.61%) were thought to be due to brucellosis, with PPR and FMD also significant causes (2.83% and 2.11%, respectively) the remainder being due to a variety of other causes.

**Table 7.** Summary of abortion and reproductive losses in the SR-study population.

Putative cause of abortion / loss	Number of cases	Percentage of total pregnant animals	Percentage of abortion cases
Brucellosis	664	7.61	45.8
PPR	247	2.83	17.1
Associated with FMD	184	2.11	12.7
Associated with poor nutrition	155	1.78	10.7
Other diseases	132	1.51	9.1
Associated with heat stress	41	0.47	2.8
Injuries and other causes	25	0.29	1.7
**Total**	**1,448**	**16.60**	**100.0**
Total number of pregnant animals	8,725		

SR, small ruminants; PPR, peste des petits ruminants; FMD, foot and mouth disease.

### General comments and responses from the farmers during the survey

All the farmers involved in this study practice extensive farm management systems. It was reported that they do not have their own specific grazing land available to them, but they each have to find grazing areas for their herds. This involves extensive migration, particularly during the dry seasons to find lush grazing areas. Overall there is a deceasing availability of pasture as the traditional grazing routes are being lost to arable farming and urban development and there is now a considerable problem of overgrazing the remaining land. Also, pastoralists passing through with their grazing herds graze on arable crops thus leading to conflicts and clashes with the crop farmers. Most of the pastoralist settlements rely on nearby streams and rivers as the only source of water and therefore face the challenges of water scarcity during the dry seasons, when most of the streams dry up. Lack of effective veterinary drugs and vaccines was also a serious management challenge mentioned by the respondents across the surveyed states.

The farmers also complained of their inability to access relevant and useful information to help them improve their farming systems, management and control of diseases. However, the group discussion revealed that farmers themselves are valuable sources of information on the epidemiology and dynamics of disease spread and maintenance amongst their herds. They have local names for diseases and can identify clinical signs associated with each disease of importance to them.

During the group discussions the participants identified possible reasons as to why they had significant losses due to mortality. These included difficulties in making correct diagnoses, insufficient availability of veterinary services (field practitioners, technicians, para-veterinarians, community animal health workers and veterinarians) who are actively engaged with the ruminant farmers and pastoralists. Also important were insufficient feed and a lack of high-quality veterinary medicines and vaccines for treatment and prevention of disease.

## Discussion

### Ranking of causes of mortalities and culling in cattle and SR

In summary there was broad agreement between the results of the farmer survey and the key informant interviews in relation to the most important causes of mortality and culling of cattle with trypanosomosis and FMD scoring the highest by both groups. Inadequate nutrition deficiency, CBPP, lumpy skin disease (LSD) and pasteurellosis were also considered responsible for major losses. These findings are supported by other sources suggesting that trypanosomosis, FMD, nutrition and CBPP are among the top five diseases causing major losses in cattle ( FMARD, 2010; Fadiga *et al*., 2013).

Also, the field survey and the key informant interviews revealed that trypanosomosis, PPR, FMD, orf and foot rot to be are amongst the top five diseases responsible for major losses in SR production. Other sources suggest trypanosomosis, PPR and orf are among the top five diseases causing major losses in SR ( FMARD, 2010; Fadiga *et al.* 2013).

Numerous published studies have identified CBPP, FMD and trypanosomosis as being the cattle diseases that have the largest negative impact on productivity in Nigeria ( Abenga *et al*., 2004; Alhaji & Babalobi 2016; Chukwuedo & Nimzing 2012; Enwezor *et al*., 2012; Gumel *et al*., 2015; Hassan *et al*., 2016; Lazarus *et al*., 2012; Majekodunmi *et al*., 2013; Okaiyeto *et al*., 2013; Shamaki *et al*., 2009; Sam-Wobo *et al*., 2010; Wungak *et al*., 2016). In the present field survey trypanosomosis was identified as the most impactful possibly because the survey was conducted amongst the pastoralist communities in Northern Nigeria and this is supported by the findings of Ezebuiro *et al*. (2009); Enwezor *et al*. (2012) and Hassan *et al*. (2016). However, at national level, CBPP is more likely to be the cattle disease responsible for the highest losses ( Okaiyeto *et al*., 2013; Gumel *et al*., 2015). Inadequate nutrition was also identified as a major problem responsible for significant impact on ruminant productivity, thereby causing economic losses to farmers. This situation might be more pronounced in the drier north of the country where there is insufficient water supply either for animals or for the all year-round provision of feed in this region. This situation has been previously described by Bolajoko *et al*. (2011) and Elelu *et al*. (2016). Brucellosis was identified as the major specific cause of abortion in ruminant species, probably *B. abortus* in cattle and *B. melitensis* in SR, as previously reported by ( Jajere *et al*., 2016; Mai *et al*., 2012; Ocholi *et al*., 2005). FMD was also considered a major cause of abortion as previously identified ( Ranjan *et al*., 2016).

### Diseases of major importance in SR

The present study revealed that trypanosomosis is a major health problem amongst SR. This appears to be a prevalent problem amongst the sheep and goat populations in the northern states of the country, where SR belonging to the pastoralists are often mixed with grazing and migrating cattle that are already infected with the disease or moving through tsetse-infested areas during grazing ( Anyaegbunam & Okafor, 2013; Ezebuiro *et al*., 2009). It is possible that this scenario might not be the same in other regions of the country where they do not practice the transhumance pastoral system.

Other diseases of importance causing major losses and low productivity amongst the SR populations were orf, FMD, CCPP, PPR and pasteurellosis. Of these five, PPR is very well known to be responsible for significant losses countrywide ( El-Yuguda *et al*., 2013; Woma *et al*., 2016). However, orf, FMD, CCPP and pasteurellosis can all be localized or spread across different regions, agro-climatic zones and even trading and grazing routes of the country ( Bolajoko *et al*., 2011; Elelu *et al*., 2016). Lack of adequate feed and water supplies have also been shown to be a major cause of mortality of SR ( Bolajoko *et al.*, 2011). Brucellosis was again revealed as the major reproductive disease responsible for losses and low productivity amongst the SR population via abortions. This finding corroborates with the results of studies conducted by Bertu (2010); Kaltungo *et al.* (2013); Ocholi *et al.* (2005); Zubairu *et al.* (2014) and Shima *et al.* (2015).

### Mortality rates

In the present study we found cattle mortality to be 15.3% and SR mortality as 30.9% of the surveyed population. It is pertinent to question whether the ‘mortality rate’ metric is a valid indicator of animal health status of a region or country. While obviously the death of an animal represents a significant and immediate loss to a farmer, non-lethal and sub-clinical disease also result in significant economic losses through decreases in productivity; these losses were not quantified in the present study. Equally animal mortality rates can be hugely variable from year to year (A R Peters, unpublished report) making it difficult to set meaningful baselines or benchmarks. We have suggested elsewhere that the mortality metric could be partitioned firstly into ‘young stock mortality’ which would tend to be more stable and repeatable and secondly into outbreaks or epizootics of disease which tend to be more episodic in nature (A R Peters, unpublished report).

Nevertheless the present study has shown that there are high losses in terms of mortality / culling due to disease among the ruminant populations of the seven states in Northern Nigeria. Undoubtedly these diseases have impacted negatively on the productivity levels of these animals, thereby resulting in economic losses for the farmers and reducing the contribution of the ruminant farming to the GDP of the nation ( Bolajoko *et al*. 2011; Elelu *et al*. 2016). In addition to the quantitative data, the farmer questionnaires identified a number of factors thought to be contributing to these losses. Among these are i) the challenges in making timely and correct diagnosis, ii) unavailability of veterinary services (particularly field practitioners, community animal health workers and para-veterinarians), iii) unavailability of efficient treatments and vaccines for prevention and iv) shortage of good quality feed, minerals, oligo-elements, vitamins and safe water.

### Reproduction losses

Rates of reproduction loss of 8.7% and 16.6% in cattle and SR, respectively, were recorded amongst the ruminant populations of the farmers involved in this study. It is notable that these rates are lower than those reported previously in the available literature ( Mai *et al.*, 2012; Zubairu *et al.*, 2014). The consensus of the key informants in this study was that these losses would be underestimated due to the practical difficulties of observing, detecting and diagnosing reproductive pathologies in the pastoralist situation, features also noted by Bolajoko *et al.* (2011) and Elelu *et al.* (2016).

### Disease ranking

The comparison of our current findings from farmers and key informants with the available literature, the extensive report by Fadiga *et al*. (2013), the field surveys and the records of the Federal Ministry of Agriculture and Rural Development ( FMARD, 2010) shows general agreement that trypanosomosis, FMD, nutritional deficiencies, CBPP and (in some cases) LSD are consistently the major diseases responsible for losses in cattle production. For SR, there is a good alignment amongst the findings from our literature review, field surveys and FMARD records that trpanosomosis, PPR and FMD are the most serious diseases responsible for major production losses. However the key informant responses were not as well aligned with the three other sources of data on SR, possibly because only few key informants responded on SR diseases. However, in general terms, the results obtained in this present study are considered to be in accordance with the disease situation for both cattle and SR in most other sub-Saharan countries.

### Other challenges

Lack of efficacious veterinary drugs was also a major management challenge mentioned by the respondents. This is similar to a previous report where the pastoralists claimed previously effective drugs that used to work were no longer effective ( Kingsley, 2015). Undoubtedly, there will be many confounding and/or contributory factors leading to this situation, which is beyond the scope of this present study. However, it is important to point out that this may be attributed to poor quality of current veterinary products in the market as was similarly reported for anthelmintic drug in other parts of Africa ( Van Wyk *et al.*, 1997). Other factors may include poor and/or long storage of drugs beyond their expiry dates, incorrect administration of drugs by livestock owners or unqualified advisers leading to treatment failure ( Bolajoko *et al.* 2011; Elelu *et al.* 2016; Kingsley, 2015).

## Data availability

### Underlying data

Harvard Dataverse: Bolajoko: Farmer Surveys and Key Informant Questionnaires on Cattle and Small Ruminant Mortality - Nigeria 2017-2018. https://doi.org/10.7910/DVN/JIRVS9 ( Bolajoko, 2020).

This project contains the following underlying data:

- 14 XLSX files containing data collected from farmer surveys by state and livestock type (cattle/small ruminants)
- 3.0 Bolajoko_Farmer_Survey_Cattle_SR_ALL_STATES.xlsx (data collected from cattle and ruminant farmer surveys for all states)
- Bolajoko_Key_Informant_Questionnaires.pdf (data collected from key informant questionnaires)

Data are available under the terms of the Creative Commons Zero "No rights reserved" data waiver (CC0 1.0 Public domain dedication).
