# Supplementary material for: Field survey of major infectious and reproductive diseases responsible for mortality and productivity losses of ruminants amongst Nigerian Fulani pastoralists
Source: Gates Open Res. 2020 Oct 19;4:162. [Version 1] doi: 10.12688/gatesopenres.13164.1 (PMC7986242; doi:10.12688/gatesopenres.13164.1)
Supplement: Peer review history

# Peer review: Reviewer response for version 1

**DOI:** 10.21956/gatesopenres.14362.r30396

**Published:** 2021-03-22

**Authors:** Shawn Babiuk

**Affiliations:** 1 National Centre for Foreign Animal Disease, Canadian Food Inspection Agency, Winnipeg, MB, Canada

The paper is well written and aims to determine which ruminant diseases impact production in Nigeria. This was achieved by using surveys for producers as well as key experts. The results identify the main diseases responsible for losses. Since the results were based on a survey there can be some bias since the diseases were not confirmed by a laboratory. This is because many diseases need to be confirmed by a diagnostic laboratory. This can be addressed in the other challenges section where there is limited veterinary laboratory capacity to perform this work. It would be interesting to see which are the main diseases diagnosed by veterinary laboratories in the country.

Is the work clearly and accurately presented and does it cite the current literature?

Yes

If applicable, is the statistical analysis and its interpretation appropriate?

Not applicable

Are all the source data underlying the results available to ensure full reproducibility?

Yes

Is the study design appropriate and is the work technically sound?

Yes

Are the conclusions drawn adequately supported by the results?

Yes

Are sufficient details of methods and analysis provided to allow replication by others?

Yes

Reviewer Expertise:

Animal disease, laboratory diagnostics

I confirm that I have read this submission and believe that I have an appropriate level of expertise to confirm that it is of an acceptable scientific standard, however I have significant reservations, as outlined above.

# Peer review: Reviewer response for version 1

**DOI:** 10.21956/gatesopenres.14362.r30400

**Published:** 2021-02-23

**Authors:** Ulf Magnusson

**Affiliations:** 1 Division of Reproduction, Department of Clinical Sciences, Swedish University of Agricultural Sciences, Uppsala, Sweden

This kind of study – to describe/map the importance and impact of livestock diseases – is indeed commendable as they help us prioritize our efforts in improving animal health and productivity and ultimately the livelihood of farmers. Here the authors describe the situation among ruminants kept by Nigerian pastoralists. One particular strength of the study is that not only the most obvious negative effect on animal health, mortality, but also another robust one reproductive failure (abortion) is recorded. The paper is well written and comprises very interesting and useful data.

 The paper would be improved by clarifying the methodological layout and some methodological details:

 The various steps and elements and interrelationship (conceptually and sequentially) in the data collection procedure is a bit tricky to follow. The group discussion with the farmers where RRA and PRA always conducted? The questionnaire-based survey among farmers is said to be starting September 2016 – is this a typo? Also, these sentences do have some mixed terminology: *The questionnaires were structured in two sections: The first covered demographic data such as age, address, duration of ownership of ruminants and species of ruminants kept. The second part focused on disease, mortality and reproductive losses. Section 2 covered questions on specific diseases.* **Then it is a description about smaller group discussion again. Perhaps some kind of flow chart would help the reader to follow the steps and procedures in the data collections.

 Did all visits/interviews that generated the quantitative data from farmers happen immediately after the one-year period in August 2018? Have the pastoralists got good records on all abortions and deaths during that year or is there a risk of recall bias? A sentence or two about this would help the reader to assess the quality of the data. Also, it would likely be more appropriate to clearly indicate that the percentages in the abstract and elsewhere are based on farmers reporting and not presented as an observational fact.

Is the work clearly and accurately presented and does it cite the current literature?

Partly

If applicable, is the statistical analysis and its interpretation appropriate?

Partly

Are all the source data underlying the results available to ensure full reproducibility?

Yes

Is the study design appropriate and is the work technically sound?

Yes

Are the conclusions drawn adequately supported by the results?

Yes

Are sufficient details of methods and analysis provided to allow replication by others?

No

Reviewer Expertise:

Animal health and reproduction in LMICs; Field surveys and questionnaire among farmers, zoonoses and antimicrobial resistance

I confirm that I have read this submission and believe that I have an appropriate level of expertise to confirm that it is of an acceptable scientific standard, however I have significant reservations, as outlined above.

# Peer review: Reviewer response for version 1

**DOI:** 10.21956/gatesopenres.14362.r30057

**Published:** 2021-01-07

**Authors:** Mark M. Rweyemamu

**Affiliations:** 1 SACIDS Foundation for One Health (SACIDS), Sokoine University of Agriculture, Morogoro, Tanzania

This is a good participatory survey that involves both key/expert informers and livestock owners/farmers. By avoiding the use of predesigned questionnaires with the farmers the authors seem to have gained confidence with the pastoral communities in teasing out responses that reflect the farmers' own concern.

 The Discussion section could be improved in the following ways:

1. Discussion Para 3 - suggest "abortions associated with FMD" - to be in line with the description in Tables 6 and 7.
2. As the whole exercise was about identifying impactful disease conditions, the authors seem to have overlooked what appear to be shared concerns for cattle and small ruminants i.e.: (a) Trypanosomosis and FMD seemed to be ranked high for both cattle and SR and also by key informants and farmers; (b) PPR was identified as the disease of specific concern for the SR while CBPP was for cattle. Such clarity would assist those who are concerned with allocating resources for coordinated control of impactful diseases.

Is the work clearly and accurately presented and does it cite the current literature?

Yes

If applicable, is the statistical analysis and its interpretation appropriate?

Not applicable

Are all the source data underlying the results available to ensure full reproducibility?

Yes

Is the study design appropriate and is the work technically sound?

Yes

Are the conclusions drawn adequately supported by the results?

Yes

Are sufficient details of methods and analysis provided to allow replication by others?

Yes

Reviewer Expertise:

infectious diseases, especially viral epidemic diseases; vaccines and one health

I confirm that I have read this submission and believe that I have an appropriate level of expertise to confirm that it is of an acceptable scientific standard.
